# Retraction: Regulation of DNA Repair Mechanism in Human Glioma Xenograft Cells both *In Vitro* and *In Vivo* in Nude Mice

**DOI:** 10.1371/journal.pone.0328089

**Published:** 2025-07-15

**Authors:** The PLOS One Editors

Following the publication of this article [1], concerns were raised about results presented in Figs 2, 3, 5, and 6. Specifically,

- There appear to be vertical irregularities suggestive of splice lines in the following panels:
  - Fig 2A, 5310 Cav-1, between lanes 2-3
  - Fig 3B, 5310 pATM, between lanes 2-3
  - Fig S2C, 4910 γH2AX, between lanes 2-3
  - Fig S2C, 4910 GAPDH, between lanes 2-3
- There appear to be regions where the area around the western blot bands does not appear to match the overall background of the following panel:
  - Fig 5D HIF-1A, lane 1
  - Fig 5D Rad51, lane 3
  - Fig 5D GAPDH, lanes 1-6
- The Fig 6C 4910 pMC DAPI panel appears to partially overlap with the Fig 6C 5310 pMU DAPI panel

The first author disagreed with the concerns raised with Figs 2, 5, and S2, and stated that the original blots underlying the results presented in this article [1] are no longer available. Regarding the concern with Fig 3, the first author stated that in the absence of the raw data they were unable to comment.

In light of the above unresolved concerns that call into question the reliability and integrity of the published results, the *PLOS One* Editors retract this article.

SP and KKV did not agree with the retraction and stand by the article’s findings. CC, DHD, and JSR either did not respond directly or could not be reached.
